# Region Met225 to Ile412 of *Bacillus cereus* Hemolysin II Is Capable to Agglutinate Red Blood Cells

**DOI:** 10.3390/molecules28083581

**Published:** 2023-04-19

**Authors:** Alexey S. Nagel, Natalia V. Rudenko, Polina N. Luchkina, Anna P. Karatovskaya, Anna V. Zamyatina, Zhanna I. Andreeva-Kovalevskaya, Alexander V. Siunov, Fedor A. Brovko, Alexander S. Solonin

**Affiliations:** 1 G.K. Skryabin Institute of Biochemistry and Physiology of Microorganisms, FRC Pushchino Scientific Centre of Biological Research, Russian Academy of Sciences, 5 Prospekt Nauki, 142290 Pushchino, Moscow Region, Russia; anagell@mail.ru (A.S.N.); paulinaluchkina@yandex.ru (P.N.L.); hemolysin6@gmail.com (Z.I.A.-K.);; 2 Pushchino Branch, Shemyakin-Ovchinnikov Institute of Bioorganic Chemistry, Russian Academy of Sciences, 6 Prospekt Nauki, 142290 Pushchino, Moscow Region, Russia; annakaratovskaya@mail.ru (A.P.K.); anna.zamjatina@yandex.ru (A.V.Z.);

**Keywords:** pore-forming toxin, monoclonal antibody, agglutination, three-dimensional protein structure modeling

## Abstract

Hemolysin II (HlyII) is one of the virulence factors of the opportunistic bacterium *Bacillus cereus* belonging to the group of β-pore-forming toxins. This work created a genetic construct encoding a large C-terminal fragment of the toxin (HlyIILCTD, M225–I412 according to the numbering of amino acid residues in HlyII). A soluble form of HlyIILCTD was obtained using the SlyD chaperone protein. HlyIILCTD was first shown to be capable of agglutinating rabbit erythrocytes. Monoclonal antibodies against HlyIILCTD were obtained by hybridoma technology. We also proposed a mode of rabbit erythrocyte agglutination by HlyIILCTD and selected three anti-HlyIILCTD monoclonal antibodies that inhibited the agglutination.

## 1. Introduction

The microorganisms *Bacillus cereus* sensu lato are spore-forming Gram-positive bacteria occurring in various ecological niches, including saprophytes and pathogens. *B. cereus* sensu scripto is a widespread opportunistic microorganism that synthesizes a number of toxins. This microorganism causes up to 12% of food poisoning—mainly gastrointestinal disease—outbreaks in the world [1]. *B. cereus* produces a wide range of virulence factors [2] and causes serious illnesses in 40% of the cases in both immunocompromised patients and premature infants [3]. Hemolysin II (HlyII) is one of the pathogenic factors of *B. cereus* and belongs to the group of β-pore-forming toxins [4]. Clinical isolates of *B. cereus* show an increased level of HlyII expression, which is indicative of its important role in the infectious process [5]. The best-known representatives of this group of toxins are *Staphylococcus aureus* α-toxin, *Clostridium perfringens* β-toxin, *B. cereus* cytotoxin K (CytK), and some others [6]. The amino acid sequence of HlyII is 38% similar to that of α-toxin *S. aureus*. The uniqueness of HlyII lies in the presence of a 94-amino acid C-terminal extension (C-terminal domain, HlyIICTD) absent in other investigated members of the β-barrel pore-forming toxin family [4,7]. It has been shown earlier that the C-terminal fragment is capable of interacting with cell membranes by oligomerization [8,9].

Previously, it has been found that a purified HlyII preparation from the recombinant *B. subtilis* BD170 strain carrying a plasmid with the complete nucleotide sequence of the structural part of the *hlyII* gene contains truncated forms at the N-terminus, which are detected by monoclonal antibodies against the C-terminal domain of HlyII [8].

Genetic coding in bacteria is largely performed by means of “one gene, one protein” paradigm. Nevertheless, structural features of mRNA, the universality of the genetic code, and the dynamic nature of translation sometimes enable organisms to deviate from the standard rules of protein coding. In particular, bacteria and bacteriophages can use several non-standard ways of translation to express more than one protein from one mRNA [10]. This provides a denser packing of genetic information in genomes. One of such alternative ways is to use additional translation initiation sites in the gene within the same reading frame [11]. Besides, it is well known that methionine residues of proteins are susceptible to oxidation by various forms of reactive oxygen species. In connection with this phenomenon, it is also necessary to take into account the possibility of post-translational modification of methionine residues with the participation of the msrAB genes, which provide further proteolysis at these residues [12,13]. One of such possible truncated peptides located within the hemolysin II gene can be important for the pore formation of this toxin as it occurs among a number of strains in which the hemolysin II gene is disrupted in various ways. A product of 188 amino acids with a mass of 21.3 kDa corresponds in size to the truncated form that exists in the lysogenic strain of *B. thuringiensis*, in which the *hlyII* gene is disrupted by an integrated bacteriophage [14]. In all strains of *B. anthracis* described so far, the *hlyII* gene has a frameshift mutation and a possible restoration of open reading frames from downstream points that potentially start either with M132 or M225 [15].

The goal of the present work was to elucidate the function of the HlyII (M225–I412) polypeptide, named HlyIILCTD, and to produce monoclonal antibodies (MAbs) against it. It was shown that HlyIILCTD was capable of agglutinating rabbit erythrocytes. The MAbs produced against HlyIILCTD were able to inhibit the agglutination.

## 2. Results

### 2.1. Isolation and Purification of Denatured and Native HlyIILCTD

**Figure 1:**
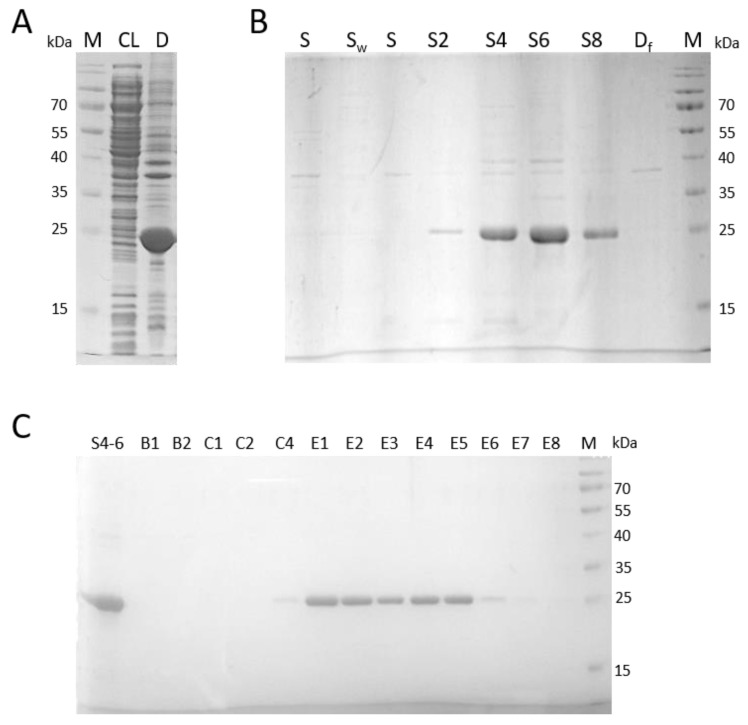
Purification of HlyIILCTD under denaturing conditions. (**A**) Analysis of HlyIILCTD expression in *E. coli* BL21 (DE3). M, protein marker; CL, clarified lysate; D, cell debris. (**B**) Analysis of protein solubility at different concentrations of urea. S, the supernatant after washing the debris with double distilled water; S_w_, the supernatant after washing the debris with buffer W; S2, the supernatant after washing the debris with 2 M urea; S4, the supernatant after washing the debris with 4 M urea; S6, the supernatant after washing the debris with 6 M urea; S8, the supernatant after washing the debris with 8 M urea; Df, residues of debris after all operations. M, protein marker. (**C**) Purification of N–TEV–LCTD from fractions S4 and S6 with Ni-NTA under denaturing conditions. S4–S6, pooled fraction from S4 and S6; B1–B2, flow through; C1–C4, fractions of buffer B wash, pH 6.3; E1–E4, fractions of elution with buffer B, pH 5.9. E5–E8, fractions of elution with buffer B, pH 4.5. M, protein marker.

The gene encoding the HlyIILCTD peptide, which was used to obtain its denatured form, was in the recombinant plasmid under the control of the T7 phage promoter. Electrophoretic analysis of the induced biomass of *E. coli* BL21 (DE3) containing the recombinant plasmid with the *hlyIIlctd* gene (Figure 1A) showed that almost all of the HlyIILCTD protein was in the debris after cell destruction. Extraction of this protein was carried out in different concentrations of urea. Figure 1B shows that the bulk of the protein is solubilized at urea concentrations of 4–8 M. Fractions solubilized in 4 and 6 M urea were pooled and used for purification by metal chelate chromatography on Ni-NTA (Figure 1C). Purified protein was found in elution fractions E1–E4 with pH 5.9 and in fraction E5, the first fraction after changing the pH of the elution buffer. The purified protein in the denatured state was used to immunize mice.

**Figure 2:**
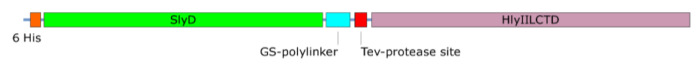
Scheme of the recombinant plasmid pTSL–HlyIILCTD.

Chaperone introduction was used to isolate and purify the native water-soluble form of HlyIILCTD. For this purpose, a fusion construct was prepared containing the *hlyIIlctd* gene and the *slyD* chaperone gene, which were separated by the TEV protease recognition site. The region encoding 6 histidine amino acid residues in this construct was coupled with the *slyD* gene and was located at the N-terminus (Figure 2).

**Figure 3:**
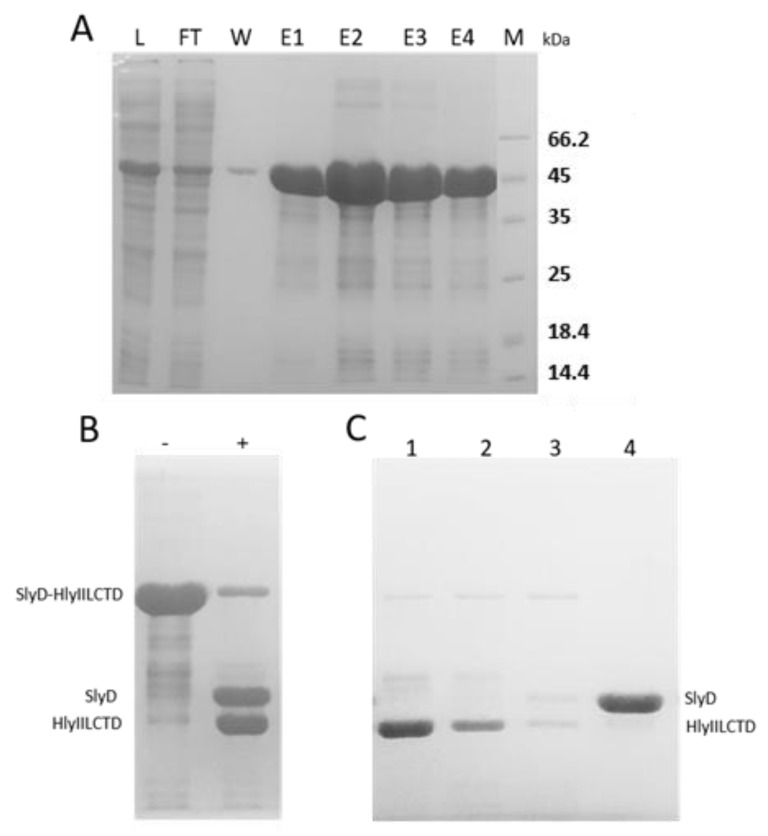
Purification of HlyIILCTD under native conditions. (**A**) Purification of the SlyD–HlyIILCTD fusion protein on Ni-NTA agarose. L, fraction of the clarified lysate of *E. coli* BL21 (DE3) pTSL–HlyIILCTD; FT, flow through; W, fraction of washing with buffer T with 50 mM imidazole; E1–E4, fractions of elution with buffer T with 300 mM imidazole; M, protein marker. (**B**) The result of proteolysis of SlyD–HlyIILCTD by TEV protease: −, protein fraction before TEV protease treatment; +, protein fraction treated with TEV protease overnight at 6 °C. (**C**) Profile of protein elution of HlyIILCTD and SlyD in a DEAE-Sepharose Fast Flow column (1–4). Elution volumes in 1.5 mL steps.

The SlyD–HlyIILCTD-fused protein was found in the clarified lysate (Figure 3A L), i.e., it was in a soluble state. At the first stage of SlyD–HlyIILCTD protein isolation, the clarified lysate was loaded onto a metal affinity column. The protein was eluted with a 300 mM imidazole buffer (Figure 3A E1–E4). Separation of HlyIILCTD and SlyD chaperone protein was performed by treating SlyD–HlyIILCTD with TEV protease (Figure 3B). Samples after proteolysis were separated in a DEAE-Sepharose Fast Flow column. The fractions eluted by the NaCl gradient were tested electrophoretically (Figure 3C). HlyIILCTD was in the flow-through fraction at 50 mM NaCl, while SlyD was eluted at 150–200 mM NaCl. Native HlyIILCTD was used for selection of MAbs and agglutination of erythrocytes.

Thus, we succeeded in purifying the recombinant protein in a native water-soluble form without any additional regions that could affect the function or immunogenicity of the protein.

### 2.2. Production of Monoclonal Antibodies against HlyIILCTD

Animals immunized with a denatured preparation of HlyIILCTD were used to obtain MAbs; immunization was performed using HlyIILCTD. Earlier, the authors had obtained a panel of MAbs against HlyIICTD [8]. When animals were immunized with the non-denaturated water-soluble form of HlyIILCTD, the sera of immune animals showed a high content of specific antibodies. However, in the process of obtaining hybridomas secreting specific antibodies, the producer clones turned out to be unstable and lost the ability to secrete antibodies. For this reason, we used animals immunized with a denatured preparation of HlyIILCTD to obtain monoclonal antibodies. MAbs that recognize a region of the molecule of HlyIILCTD other than HlyIICTD were selected against non-denaturated water-soluble HlyIILCTD. Immune sera were characterized not only by the titer (the relative content of antibodies that specifically bind to the antigen) but also by the competitive enzyme immunoassay (EIA) to determine antibodies that interact directly with regions of HlyIILCTD. During the competitive EIA, the binding of immune sera to the antigen at a point located in the linear titration range was inhibited by an excess of HlyIILCTD. For hybridization and subsequent production of hybridomas secreting MAbs with a given specificity, monoclonal antibodies recognizing only the HlyIILCTD fragment had been selected. As a result, a serum whose binding to the immobilized antigen in the EIA was inhibited by an excess of HlyIILCTD at a dilution of 1/16,000 by 71% was selected. A high serum titer indicated the formation of a sufficient pool of plasma cells to obtain hybridoma cell lines that stably produce MAbs. Splenocytes and cells of the popliteal lymph nodes were used as a source of lymphocytes for hybridomas secreting MAbs against HlyIILCTD according to the method [16]. Hybridoma cell lines secreting MAbs were obtained by the fusion of lymphocytes and myeloma cell line SP2/0 with polyethylene glycol. Selection of hybridomas secreting specific antibodies was performed by indirect EIA by interaction of supracellular supernatants with soluble HlyIILCTD immobilized on immunoplates. Clones showing a positive signal were further analyzed for interaction with HlyIICTD. Based on the assessment of proliferative activity and stability of antibody production, four stable hybridoma clones were selected that secreted MAbs against HlyIILCTD. All obtained antibodies contained the κ light chain (kappa); MAbs LCTD-70 and LCTD-75 contained the γ1 heavy chain; LCTD-71 and LCTD-76, γ2a.

### 2.3. Agglutination of Erythrocytes and Inhibition of Agglutination by Antibodies against HlyIILCTD

**Figure 4:**
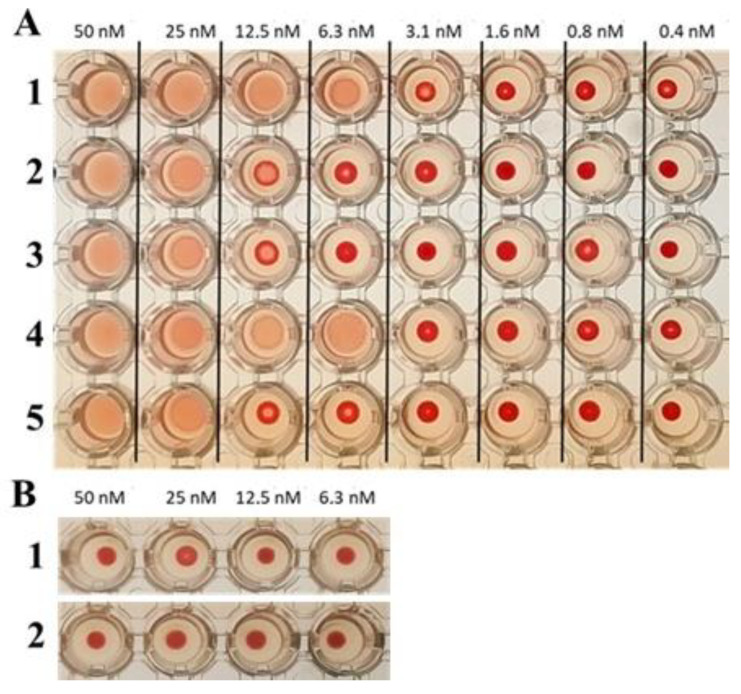
(**A**) Inhibition of agglutination of a 0.5% suspension of rabbit erythrocytes by monoclonal antibodies against HlyIILCTD. The upper line shows the concentration of HlyIILCTD in each column; 1, agglutination in the presence of HlyIILCTD; 2, agglutination in the presence of HlyIILCTD and 0.5 μM MAb LCTD-70; 3, agglutination in the presence of HlyIILCTD and 0.5 μM MAb LCTD-71; 4, agglutination in the presence of HlyIILCTD and 0.5 μM MAb LCTD-75; 5, agglutination in the presence of HlyIILCTD and 0.5 μM MAb LCTD-76. (**B**) The upper line shows the concentration of SlyD in each well; 1, in the presence SlyD; 2, in saline.

The HlyIILCTD preparation incubated with rabbit erythrocytes caused their agglutination, which indicated its ability to bind to the cell surface. Agglutination of a 0.5% suspension of rabbit erythrocytes was observed up to a concentration of about 6.3 nM (Figure 4). Agglutination was reversible, did not lead to erythrocyte lysis, and erythrocytes retained their integrity, which was confirmed by the absence of changes in the adsorption of the supernatant of erythrocytes at a wavelength of 541 nm before and after the agglutination reaction. Figure 4B shows controls demonstrating the absence of erythrocyte agglutination in the presence of SlyD, as a possible impurity contained in the preparation, and saline. A 94-amino acid C-terminal excess of HlyII did not cause agglutination, which indicates the involvement (in agglutination) of the hemolysin II region located between the C-terminal domain and the major part of the toxin [9].

The addition of MAbs against HlyIILCTD (LCTD-70, LCTD-71 and LCTD-76) to the reaction mixture resulted in the suppression of the agglutination of rabbit erythrocytes at a 40-fold molar excess. MAb LCTD-75 under these conditions did not inhibit the agglutination of rabbit erythrocytes.

## 3. Discussion

Previously, analysis of purified forms of HlyII using affinity chromatography on a Ni-NTA column has revealed proteins truncated at the N-terminus, which are effectively bound by monoclonal antibodies [8], since their open reading frames coincide with the frame of the *hlyII* gene. Among the identified truncated forms, a product of 188 amino acids with a mass of 21.3 kDa—which corresponds in size to HlyIILCTD and begins with M225—has been detected [8]. The same form can exist both in the lysogenic *B. thuringiensis* strain [14] and in all strains of *B. anthracis* described so far [15]. Thus, it can be assumed that a protein product starting with M225 can exist and have a possible functional load in the bacterial cell.

Analysis of the representativeness of the hemolysin II gene among the genomes of *B. cereus*, *B. anthracis*, and *B. thuringiensis* using the PATRIC Comparative Systems service detected truncated forms of this gene. Strains containing the hemolysin II gene with an interrupted coding sequence were selected and combined into a group, the sequences of which were aligned using PATRIC MSA. Most representatives of *B. thuringiensis* with a disturbed *hlyII* open reading frame contain a sequence that potentially encodes the region M225–I412, which we called HlyIILCTD (Table 1). All representatives of *B. cereus* sensu scripto containing the *hlyII* gene have no abnormalities in this gene, except for one strain in which the region encoding the signal sequence is impaired (Table 1). All members of *B. anthracis* contain a disrupted *hlyII* but possess a sequence potentially encoding M132–N371 (Table 1). Thus, bioinformatic analysis showed that all hemolysin II genes with an interrupted coding sequence and incapable of synthesizing a full-length toxin contained a coding sequence that partially or completely included HlyIILCTD. The retention of the truncated open reading frame of the *hlyII* gene suggests the functional significance of HlyIILCTD as an independent protein product.

**Figure 5:**
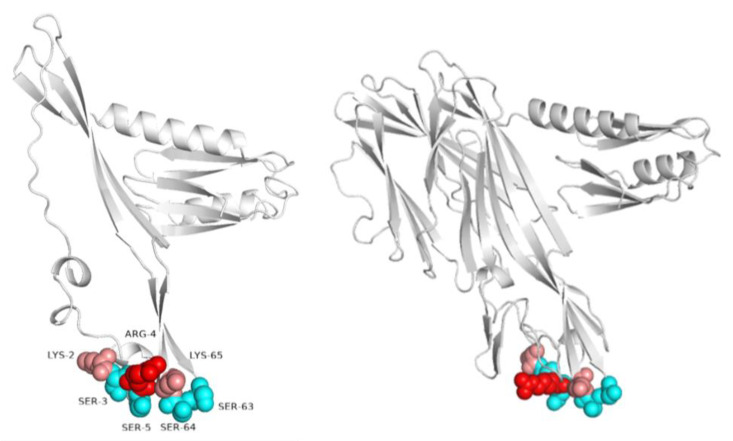
Proposed site of HlyIILCTD binding to erythrocyte-surface glycosaminoglycans. On the left, the HlyIILCTD structure predicted by AlphaFold [20,21] for a putative protein from *B. thuringiensis* ser. *israelensis* ATCC 35646 Uniprot: Q3EPX6. On the right, the structure of the full-length HlyII toxin from *B. cereus* ATCC 14579 Uniprot: Q81AN8, predicted by AlphaFold [20,21]. The putative GAG-binding site is highlighted in color. Positively charged amino acids lysine and arginine are shown in red and salmon color; serines are highlighted in cyan.

Cell agglutination is mainly determined by the interaction of the cell surface with charged groups of proteins that provide adhesion. The binding sites in such proteins always contain the charged amino acids lysine and arginine, the positive charges of which presumably interact with the negatively charged sulfates and carboxylates of the glycosaminoglycan (GAG) chains [17]. In [18], it is suggested that the consensus sequences for glycosaminoglycan recognition are defined as [-X-B-B-X-B-X-] and [-X-B-B-B-X-X-B-X-], where B is arginine or lysine and X is any amino acid. Amino acids such as serine and glycine also play an important role in the interaction of the protein with GAGs, providing good flexibility of the binding site and minimal steric restrictions due to small side chains [19]. In many cases, the structure of the GAG binding site includes loops that make its positioning more variable [17]. The structure of HlyIILCTD Uniprot: Q3EPX6 predicted by AlphaFold [20,21] has a spatial region of Lys2 (Lys226 in full-length HlyII), Ser3, Arg4, Ser5, Ser63, Ser64, and Lys65, consisting of positively charged amino acids and serines, the structure of which also includes loops and, in terms of the spatial arrangement of amino acids, is homologous to the consensus sequence [-X-B-B-X-B-X-] proposed in [18] (Figure 5).

It can be assumed that HlyIILCTD is capable of causing erythrocyte agglutination by binding to surface glycosaminoglycans through this spatial region. On the structures of HlyIILCTD and full-length HlyII predicted by AlphaFold [20,21] (Figure 5), these amino acids are on the surface, and it is likely that the full-length HlyII toxin can also interact with GAGs and provide erythrocyte agglutination. The process of hemolysis can occur simultaneously with or ahead of erythrocyte agglutination. The fact of agglutination of erythrocytes by a toxin fragment, which was first shown by the authors, can be important in the functioning of the toxin.

**Figure 6:**
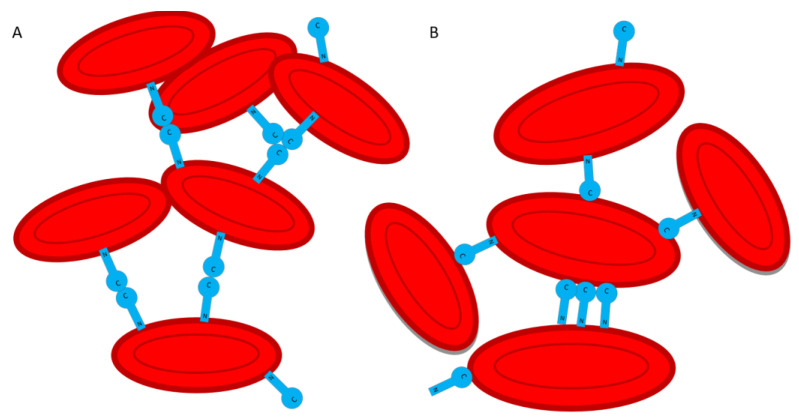
Proposed schemes of erythrocyte agglutination. (**A**) Agglutination can occur due to the binding of the N-terminal region of HlyIILCTD to the surface of erythrocytes and the oligomerization of the C-terminal region of HlyIILCTD. (**B**) Agglutination can occur because of the binding to the surface of both the N-terminal and C-terminal regions of HlyIILCTD.

Apparently, agglutination of erythrocytes in the presence of HlyIILCTD occurs due to the presence of two functional sites. We have previously shown that HlyIICTD is capable of binding to erythrocyte membranes [8] and of effectively oligomerizing in their presence [9]. There are at least two possible mechanisms of erythrocyte agglutination caused by HlyIILCTD. In the first case, the binding occurs due to a site located on HlyIILCTD, which interacts with the carbohydrate component of the cell surface, and its C-terminal part is involved in the oligomerization of HlyIILCTD (Figure 6A). In the second, agglutination can occur due to the binding of both the N-terminal and C-terminal regions [9] of HlyIILCTD (Figure 6B).

Studying the effect on the efficiency of agglutination of MAbs against HlyIILCTD, we found that three MAbs (LCTD-70, LCTD-71 and LCTD-76) were able to suppress its manifestation and one MAb (LCTD-75) did not affect erythrocyte agglutination. It is possible that the epitope of the latter is localized in such a way that its interaction with the MAb does not prevent agglutination, while the interaction of the three MAbs with their epitopes in HlyIILCTD hinders it.

## 4. Materials and Methods

### 4.1. Cloning of HlyIILCTD

The gene encoding HlyIILCTD *B. cereus* ATCC 14579, starting with methionine at position 225 (M225–I412), was amplified from genomic DNA using the primers:

F_LCTD_N: 5′-TAATACATATGAAATCACGTAGTTATAATGAAGG

R_LCTD_X: 5′-TAATACTCGAGTCAGATCTGTTTAATCTCGATA

**Figure 7:**
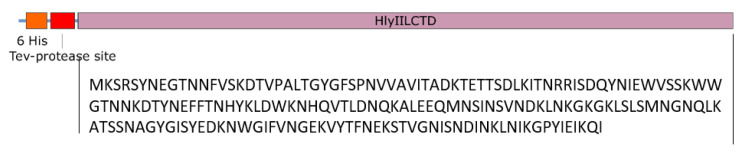
Amino acid sequence of HlyIILCTD from *B. cereus* ATCC 14579 (M225-I412, according to the full length of the HlyII toxin [7]).

The PCR product was cloned with pET19mod [21] at the NdeI and XhoI sites under the control of the T7 phage promoter. Recombinant plasmids were transformed into the expression strain *E. coli* BL21 (DE3). The amino acid sequence of HlyIILCTD confirmed by DNA sequencing is shown in Figure 7.

The culture was grown at 37 °C in 2 flasks of an LB medium (150 mL each), containing ampicillin at a concentration of 100 μg/mL, to OD600 = 0.6–0.8; then the expression of HlyIILCTD was induced by adding isopropyl-β-d-1-thiogalactopyranoside to a final concentration of 0.1 mM. The cultivation proceeded further at 32 °C for 12 h. Biomass was collected on an Eppendorf 5810 R centrifuged at 6000× *g* rpm for 15 min, resuspended in 3 mL of buffer A (50 mM NaH_2_PO_4_, 500 mM NaCl, 5% glycerol, pH 8) treated with 1 mM phenylmethylsulfonyl fluoride and 0.1% lysozyme. The resulting cell suspension was sonicated on a QSonica Q700 ultrasonic homogenizer (7 cycles of 20 s each with 2 min breaks; amplitude, 35%). Disrupted cells were centrifuged on an Eppendorf MiniSpin centrifuge for 30 min at 13,400 rpm at 6 °C. The resulting clarified lysate and cell debris were analyzed by SDS-PAGE. For further purification of HlyIILCTD, cell debris was washed step by step with 2 mL of bidistilled water, 2 mL of buffer W (50 mM Tris-HCl, 0.5% Triton X-100, 1mM EDTA, pH 8), again with 2 mL of bidistilled water, and then with 2 mL of buffer B (100 mM NaH_2_PO_4_, 10 mM Tris-HCl, pH 8) containing 2 M (fraction S2), 4 M (fraction S4), 6 M (fraction S6) and 8 M (fraction S8) urea. Fractions S4 and S6 were pooled and used for purification by Ni-NTA metal chelate chromatography (Qiagen, Germantown, TN, USA) according to the manufacturer’s protocol. The purified denatured protein was used to immunize mice.

### 4.2. Expression and Purification of HlyIILCTD under Native Conditions

The gene encoding the HlyIILCTD region of *B. cereus* ATCC 14579 was amplified using the primers

F_LCTD_B: 5′-TAATAGGATCCATGAAATCACGTAGTTATAATG

R_LCTD_X: 5′-TAATACTCGAGTCAGATCTGTTTAATCTCGATA

and cloned using the pTSL vector [22] at the BamHI and XhoI sites. The resulting pTSL–HlyIILCTD construct was confirmed by DNA sequencing. The amino acid sequence of the target product encoded in pTSL–HlyIILCTD consisted of HlyIILCTD fused through the TEV protease site to the SlyD chaperone with six histidine residues at the N-terminus (Figure 2).

An overnight culture of strain *E. coli* BL21 (DE3) transformed with the pTSL–HlyIILCTD plasmid was diluted 50-fold in an LB medium containing 100 μg/mL ampicillin (2 flasks of 200 mL each) and was grown at 37 °C to OD600 = 0.5–0.6 with intensive aeration. Expression of SlyD–LCTD was induced by adding isopropyl-β-d-1-thiogalactopyranoside to a final concentration of 0.1 mM. Further cultivation continued at 20 °C for 12 h with intensive aeration. Upon induction, cells were precipitated on an Eppendorf 5804 R centrifuged in 50 mL tubes (Grainer, Kremsmünster, Austria) at 6000× *g* rpm for 15 min; the precipitate was stored at −20 °C. To isolate the SlyD–HlyIILCTD protein, cells harvested from 400 mL of culture were resuspended in 30 mL in ice with buffer T (50 mM Tris-HCl, 300 mM NaCl, 5% glycerol, pH 7.5) with 20 mM imidazole and sonicated on an S-4000/CL5 Misonix Ultrasonic Liquid Processor (8 cycles of 30 s each; amplitude, 35%; 3 min breaks). The resulting cell lysate was centrifuged for 1 h at 12,000 rpm in an Avanti JXN-26 centrifuge. The clarified lysate was used for further purification in several steps. Metal affinity chromatography was used to isolate HlyIILCTD fused with SlyD chaperone. An amount of 30 mL clarified lysate was applied to a 1 mL column of Ni-NTA agarose (Qiagen, Germantown, TN, USA) equilibrated with buffer T containing 20 mM imidazole. Upon application of the preparation, the column was washed with 10 volumes of buffer T with 20 mM imidazole and 10 volumes of buffer T with 50 mM imidazole. The protein was eluted with buffer T containing 300 mM imidazole. To separate HlyIILCTD and SlyD protein, the SlyD–HlyIILCTD preparation was treated with TEV protease. An amount of 180 μg of TEV protease isolated according to [23] was added to 3 mg of the SlyD–HlyIILCTD preparation. Proteolysis was carried out during the dialysis for 24 h at 6 °C. The resulting reaction mixture was dialyzed in 2 steps. The first step was against a buffer containing 50 mM Tris-HCl, pH 7.5, 300 mM NaCl, 1 mM EDTA, pH 8.0, 5 mM 2-mercaptoethanol, 5% glycerol; the second step was against a buffer containing 50 mM Tris-HCl, pH 7.5, 300 mM NaCl, 5 mM 2-mercaptoethanol, 5% glycerol. The samples after proteolysis were applied to a DEAE-Sepharose Fast Flow column (Pharmacia, Uppsala, Sweden) equilibrated with the same buffer. The proteins were eluted with a NaCl gradient from 0.05 to 1 M in the same buffer in a volume of 20 mL; the fractions were tested electrophoretically. Native HlyIILCTD was used for selection of MAbs and agglutination of erythrocytes.

### 4.3. Production and Isolation of MAbs

To obtain hybridomas secreting MAbs, we used animals kept under standard conditions in accordance with the Decree of the Chief State Sanitary Doctor of the Russian Federation dated 29 August 2014 No. 51 “On approval of SP 2.2.1 ‘The maintenance of experimental biological clinics (vivariums)’”. Female BALB/c mice aged 2–3 months were immunized four times with 10 μg HlyIILCTD (per animal) with an interval of 2 weeks. Seven days after the last injection, blood was taken from the caudal vein, and the content of specific antibodies was assessed by indirect EIA according to [8] in order to choose an animal with a higher titer. Booster immunization was performed after 1 month. Three days before cell fusion, 10 μg HlyIILCTD in PBS was injected subcutaneously into each animal’s paws and intraperitoneally. Hybridomas secreting MAbs were obtained by hybridoma technology [16] with modifications [24].

MAbs were isolated by affinity chromatography on Protein A Sepharose (Thermo Fisher Scientific, Waltham, MA, USA) [25] from culture fluids of hybridomas secreting these MAbs. The types of heavy and light immunoglobulin chains were determined by EIA using the Rapid ELISA Mouse MAb Isotyping kit (Thermo Fisher Scientific, Waltham, MA, USA) according to the manufacturer’s instructions.

### 4.4. Agglutination of Erythrocytes and Inhibition of Agglutination by Antibodies

The efficiency of HlyIILCTD agglutination was evaluated by diluting the test drug in a suspension of rabbit erythrocytes. The drug was preliminarily titrated in steps of two in 50 µL of saline in a U-bottom 96-well microplate followed by the addition of an equal volume of 1% erythrocyte suspension. The plate was incubated at room temperature for 2 h. To assess the effect of MAbs on agglutination, antibodies, and HlyIILCTD were preincubated for 15 min at room temperature, after which erythrocytes were added to a final concentration of 0.5%. The final antibody concentration was 0.5 μM at the titration of HlyIILCTD in steps of two from 50 nM to 0.4 nM. During the observation period, we noted the gradual appearance of a red-colored precipitate consisting of erythrocytes at the bottom of the U-well in the presence of HlyIILCTD. The result was recorded after 2 h of incubation in a U-bottom 96-well microplate at room temperature.

### 4.5. Bioinformatic Analysis

Sequence analysis of HlyIILCTD was performed using the PATRIC service [26].

## 5. Conclusions

The large C-terminal fragment of HlyII (M225–I412) causes agglutination of rabbit erythrocytes. Among the MAbs against HlyIILCTD, three were found, the addition of which suppressed erythrocyte agglutination.

## Figures and Tables

**Table 1:** Representativeness of the *hlyII* gene among members of the *B. cereus* sensu lato.

Species:	*Bacillus anthracis*	*Bacillus thuringiensis*	*Bacillus cereus*
number of analyzed strains	51	82	132
*hlyII* gene is completely missing	0	34	80
*hlyII* gene is interrupted	51	8	1
*hlyII* gene is not broken	0	40	51

## Data Availability

Not applicable.

## Sample Availability

Samples of the compounds are available from the authors.
